# The similarity between arbuscular mycorrhizal fungi communities of trees and nearby herbs in a planted forest exhibited within-site spatial variation patterns explained by local soil conditions

**DOI:** 10.1007/s00572-025-01197-5

**Published:** 2025-03-14

**Authors:** Akotchiffor Kevin Geoffroy Djotan, Norihisa Matsushita, Yosuke Matsuda, Kenji Fukuda

**Affiliations:** 1 Graduate School of Agricultural and Life Sciences, University of Tokyo, Bunkyō, Japan; 2 Graduate School of Bioresources, Mie University, Tsu, Japan

**Keywords:** AMF ecology, Forest ecosystem, Common AMF, Host plant life form, Next-generation sequencing

## Abstract

The similarity between the arbuscular mycorrhizal fungi (AMF) communities of trees and neighboring understory herbs in forests remains unclear, which we aimed to clarify. We traced and collected basal roots of 20 randomly chosen *Cryptomeria japonica* (Cupresaceae) trees and the surrounding soil at four microsites in 1 km^2^ of a *Cr. japonica* forest. One *Chloranthus serratus* (Chlorantaceae) herb immediately at the base of each sampled tree was excavated to collect an intact root system. We amplified a partial small subunit of fungal ribosomal DNA (18S) using Illumina MiSeq amplicon sequencing. Soil physicochemical properties were also measured. We detected 670 and 679 AMF operational taxonomic units (OTUs) in *Cr. japonica* and *Ch. serratus*, respectively, belonging to *Acaulospora*, *Dominikia*, *Glomus*, *Microkamienskia*, *Rhizophagus*, *Septoglomus*, and *Sclerocystis*. Seventeen OTUs were detected in the roots of both host species at average relative abundances > 1%. Among them, four dominant OTUs with an average relative abundance > 10% were concurrently detected in the roots of 17 tree–herb sets. The composition and similarity of their AMF communities were spatially varied, significantly driven by spatially varying soil pH, total C, N, C/N, and elevation, but not electroconductivity, supported by the microsite-dependent distributions of their dominant OTUs. We concluded that the similarity of AMF communities between trees and neighboring understory herbs depends on the soil physicochemical conditions that influence the distribution of their dominant AMF.

**Supplementary Information:**

The online version contains supplementary material available at 10.1007/s00572-025-01197-5.

## Introduction

According to recent studies and observations of mycorrhizal networks in experimental settings, arbuscular mycorrhizal fungi (AMF) connect neighboring plants of the same or different species underground, potentially contributing to nutrient exchange between individual plants (Giovannetti et al. 2004; Mikkelsen et al. 2008; Barto et al. 2012; Wipf et al. 2019; Karimi-Jashni and Yazdanpanah 2023; Merckx et al. 2024). In a forest ecosystem, AMF colonize multiple plants and plants are colonized by multiple AMF making the belowground mycelial network complex (Bunn et al. 2024). Three types of mycorrhizal interaction networks, i.e. hyphal network, bipartite mycorrhizal interaction network, and common mycorrhizal network, and the differences between them have been clarified by Karst et al. (2023). However, due to methodological reasons, it is difficult to study hyphal and common mycorrhizal networks of AMF. In the meantime, bipartite mycorrhizal interaction network which would consist in checking the presence and absensce of a given taxonomic unit of interest without confirmation of physical connections, is commonly used in natural habitats to infer the potential interactions between different functional groups of soil and plant microorganisms (Deng et al. 2012; Toju et al. 2014a, b; Chen et al. 2017).

Based on the previous evidences that nutrient might move between plants in a natural environment through mycorrhiza (Teste et al. 2009; Simard et al. 2015; Merckx et al. 2024), it is obvious that some AMF simultaneously colonize neighboring plants, but we do not know whether these neighboring plants associate with similar AMF communities. Unfortunately, little interest has been accorded to the similarity of the root colonizing AMF communities of neighboring plants (comprising tree–tree, tree–herb, and herb–herb) in forest ecosystems. To date, only tree–tree (Djotan et al. 2023) and herb–berb (Torrecillas et al. 2012) combinations have been partially investigated. Thus, studying the similarity between the root AMF communities of a tree and its neighboring herb plants will add an important ecological information to what we currently know about AMF community ecology.

AMF communities associated with the woody plants *Cryptomeria japonica* (Zou et al. 2021; Matsuda et al. 2021; Djotan et al. 2022, 2023, 2024a, b) and *Cunninghamia lanceolata* (Lu et al. 2019, 2022; Cao et al. 2020; Lin et al. 2020; Wu et al. 2022; Han et al. 2024), both belonging to Cupressaceae, have been extensively investigated in Japan and China. However, none of these studies have addressed their similarities with AMF communities in the roots of their neighboring understory herbs. *Chloranthus serratus* (Chloranthaceae) is an understory perennial plant that grows well in some *Cr. japonica* plantations in Japan. This herb has anti-inflammatory properties and is important in Chinese medicine (Zhang et al. 2012; Sun et al. 2019). However, although it is expected to form arbuscular mycorrhizae, there are no records of its mycorrhizal status, and its associated AMF community remains unknown (Wang and Qiu 2006). These two plant species offer the opportunity to fill an important gap of knowledge in the ecology of plant–soil–AMF interactions.

In this study, we aimed to clarify the similarities of the AMF community between the roots of trees and that of nearby herbs. Because *Cr. japonica* and *Ch. serratus* grow in proximity and their roots are densely interwoven (Online Resource 1), we hypothesized that they associate with similar AMF communities when growing adjacent to each other. To test this hypothesis, we used molecular approaches based on Illumina’s next-generation amplicon sequencing to investigate the AMF assemblages in neighboring tree–herb sets of *Cr. japonica* and *Ch. serratus* at four microsites within an area of 1 km^2^ of a *Cr. japonica* forest in central Japan.

## Material and methods

### Study site and sampling design

The study site was a planted forest of *Cr. japonica* in the cool-temperate region of Japan (Saitama Prefecture, 138.83°E, 35.94°N). The surrounding vegetation of the plantation is a naturally regenerated forest composed of deciduous hardwoods. The mean annual temperature and mean annual precipitation are 11.2 °C and 1,498 mm, respectively. In winter, snowfall piles to a depth of 20–30 cm. The stand density at the time of sampling was 1,050 trees/ha. The understory vegetation was mainly composed of *Amphicarpaea edgeworthii* (Fabaceae), *Ch. serratus*, and *Viola tokubuchiana* var*. takedana* (Violaceae) (Djotan et al. 2023).

In August 2022, we collected roots and surrounding soils of *Cr. japonica* trees and *Ch. serratus* herbs at four microsites (MSs 1, 2, 4, and 5) in permanent plots previously established at the University of Tokyo Chichibu Forest (Djotan et al. 2024b, Online Resource 2). The microsites were established along two topographic profiles, where MSs 1 and 4 were located at elevations of approximately 1,030–1,045 m a.s.l. (Table 1). MS2 (same topographic profile as MS1) and MS5 (same topographic profile as MS4) were located at elevations of approximately 900 m a.s.l.. At each microsite, five trees of *Cr. japonica* were arbitrarily selected. A basal root of each tree was traced from the base of the tree and an average of 50 g fresh roots with sufficient numbers of first- and second-order fine roots was sampled without disturbinng the root’s architecture (Djotan et al. 2022). Then, one *Ch. serratus* herb growing immediately at the base of the sampled tree was arbitrarily selected and excavated from the soil to collect the entire root system. Because of the proximity between paired trees and herbs, soil samples were collected from the base of the trees only for each plant pair, and placed with the roots into a labeled plastic bag. Five sets of plants (tree–herb) were collected at each microsite, resulting in 40 root and 20 soil samples. The samples were stored at 4 °C and processed within 3 days of collection.

**Table 1:** Summary of sampling site and soil physicochemical properties

Microsites	Longitude (°E)	Latitude (°N)	Elevation (m)	EC(µS/Cm)	pH	TC (%)	TN (%)	C/N
MS1	138.8252064	35.94486857	1045.7 ± 6.6 a	270.2 ± 87.5 a	5.9 ± 0.1 a	11.8 ± 2.8 b	0.7 ± 0.1 b	16.3 ± 1.0 a
MS2	138.825972	35.94276414	896.6 ± 11.4 c	269.8 ± 33.4 a	5.2 ± 0.3 b	11.3 ± 0.5 b	0.8 ± 0.0 b	15.0 ± 0.4 b
MS4	138.8240636	35.94447363	1033.6 ± 6.1 a	249.6 ± 42.5 a	5.0 ± 0.3 b	16.6 ± 2.1 a	1.0 ± 0.1 a	16.6 ± 0.5 a
MS5	138.8234634	35.94243657	909.5 ± 5.4 b	313.0 ± 54.8 a	5.2 ± 0.1 b	16.4 ± 0.6 a	1.0 ± 0.0 a	16.7 ± 0.2 a

Five pairs of *Cryptomeria japonica* and *Chloranthus serratus* were collected from each microsite (MS). Values are presented as average ± SD (*n* = 5) for elevation, soil EC (electroconductivity), pH, TC (total C), TN (total N), and C/N (carbon to nitrogen ratio). For each variable (in columns), groups (rows) with the same letter are not significantly different according to the analysis of variance followed by the Tukey HSD test (EC, pH, TC, and TN) and the Kruskal–Wallis rank sum test followed by a post hoc analysis based on the Bonferroni adjustment method (C/N) at a confidence level of *p* < 0.05 (Table S1)

### Soil physicochemical properties

Soil samples were air-dried and passed through a 500-µm mesh sieve before physicochemical analysis. Soil electroconductivity (EC) was determined using a compact conductivity meter (LAQUAtwin-EC-33; Horiba, Kyoto, Japan) and pH was measured using a compact pH meter (LAQUAtwin-pH-33; Horiba), following the procedures described in Djotan et al. (2023). Powdered soil samples (20 mg) were dry-combusted in an automatic highly sensitive NC analyzer (SUMIGRAPH NC-22F; Sumika Chemical Analysis Service, Ltd., Tokyo, Japan) that had been calibrated with the primary acetanilide standard following the manufacturer’s instructions to analyze total C, and N contents. Then, C/N ratio was calculated and used as a measure of the soil physicochemical properties (Djotan et al. 2024a).

### DNA extraction and amplification

DNA extraction and polymerase chain reaction (PCR) were performed as previously described in Djotan et al. (2024a). Briefly, lyophilized root samples were milled, and DNA was extracted using a DNeasy Plant Mini Kit (Qiagen, Germantown, MD, USA) or Extrap Soil DNA Kit Plus (Nippon Steel & Sumikin Eco-Tech. Co., Tokyo, Japan), according to the manufacturer’s instructions. We used the Extrap Soil DNA Kit Plus for six root samples because the DNeasy Plant Mini Kit failed to extract total DNA from them. We amplified approximatively 550 bp of the partial rubilose biphosphate carboxylase large subunit (*rbc*L) region using the primers rbcLaF (5′-ATG TCA CCA CAA ACA GAG ACT AAA GC-3′) (Hasebe et al. 1994) and rbcLaR (5′- GTA AAA TCA AGT CCA CCR CG-3′) (Ferri et al. 2015) to confirm the root identity and excluded inappropriate samples (Djotan et al. 2022). PCR for the amplification of approximately 550 bp of the fungal small subunit ribosomal fungal DNA (SSU rDNA) was performed on the identity-confirmed samples, as described by Djotan et al. (2024a). Briefly, primer sets AML1/AML2 (Lee et al. 2008) and NS31/AM1 (Simon et al. 1992; Helgason et al. 1998) were used in nested PCR. The Illumina adapters Tn5ME A and Tn5ME B were linked to the primer set NS31/AM1, and the purified PCR products were multiplexed by pools of five samples each. After purification, the amplicons of *rbc*L and SSU rDNA were sent to Macrogen Japan (Tokyo, Japan) for Sanger (~ 550 bp) and Illumina MiSeq (2 × 300 bp) amplicon sequencing, respectively.

### Bioinformatic and phylogenetic analyses

The Sanger sequences of the *rbc*L region were visualized for quality control and BLASTed against the National Center for Biotechnology Information (NCBI) GenBank database to confirm that the processed root samples contained *Cr. japonica* and *Ch. serratus* as expected. We processed the Illumina MiSeq amplicon sequences using QIIME 2 v. 2022.2.0 (Bolyen et al. 2019). Briefly, we demultiplexed the reads and merged the pairs to discard the forward and reverse reads that could not be merged successfully. The merging allowed an overlap of at least 10 bp and merged sequence sizes between 500 and 600 bp. Next, we filtered the merged sequences based on the q-score over a quality window of 3 bp along the sequences to keep those with a Phred quality score ≥ 20, and a minimum length fraction of 0.75. The resulting denoised sequences were clustered into operational taxonomic units (OTU) at a 97% sequence similarity threshold, excluding rare (< 10 reads across all samples or detected in only one sample) and chimeric OTUs. The retained OTUs were locally BLASTed against the Maarj*AM* (Öpik et al. 2010) and NCBI GenBank databases using the NCBI-blast-2.10.0 + program (Morgulis et al. 2008). Further BLAST searches were carried out against the GlobalAMFungi on the web platform (Větrovský et al. 2023). After this step, the few remaining non-Glomeromycotina OTUs were manually discarded. Taxa were assigned to OTUs based on the best matches in the databases, but taxonomic affiliations were kept only when query cover and percent identity to the best match were both > 95%. We updated the recovered taxonomic information following the consensus AMF classification (Redecker et al. 2013). We deposited the sequence read archives at the NCBI (PRJNA898865). The representative nucleotide sequences of the AMF OTUs generated (OR740684–OR741366, SUB13934051) and a representative partial nucleotide sequence of *rbc*L for *Cr. japonica* (OP832014, BankIt 2642437) and *Ch. serratus* (OP832018, BankIt 2642437) were submitted to GenBank.

Using MAFFT v7.490 (Katoh and Standley 2013), we aligned the representative sequences of the major OTUs, defined as those with an average relative abundance > 1% at the study site, and performed a phylogenetic analysis for taxa assignment. We inferred their maximum-likelihood phylogenetic positions using an automatic model finder and tested them with ultrafast bootstraps (UFBoot) over 1000 randomizations implemented in IQ-TREE 2 (Minh et al. 2020). We relied on a clade only when its associated UFboot ≥ 95%. The tree was annotated and displayed using FigTree v.1.4.4 (http://tree.bio.ed.ac.uk/software/figtree/). Major OTUs with an average relative abundance of at least 10% at the site or a microsite were defined as the dominant AMF of the host plant.

### Statistical analysis

All statistical analyses were performed using R v.4.3.2 with a confidence level of 0.05 (R Core Team 2023) and all permutational analyses were performed with 1000 replicates. Spatial variations in soil physicochemical properties (variation between microsites) were tested using a one-way analysis of variance (ANOVA) followed by Tukey’s honestly significant difference (HSD) when the conditions for parametric tests (normality of distribution and equality of variance) were met. Otherwise, we used the Kruskal–Wallis’ rank sum test followed by post hoc analysis based on the Bonferroni adjustment method to test variations and compare the mean values of variables between microsites. Using a two-way ANOVA (factors were microsite and host plant species), we compared the OTU richness and Shannon index of the AMF communities between the host plant species (*Cr. japonica* and *Ch. serratus*) and microsites (MSs 1, 2, 4, and 5) and tested the variation in these diversity indexes for each host plant species among microsites. AMF communities were ordinated using non-metric multidimensional scaling (NMDS) on the Bray–Curtis dissimilarity matrix computed on the relative abundance of OTUs in each sample. We visualized the ordination to analyze potential variations between microsites and host plant species, which were tested with a permutational ANOVA (PERMANOVA) using *adonis2* of vegan package. We analyzed the similarity (ANOSIM) between AMF communities in the roots of *Cr. japonica* and *Ch. serratus* at each microsite using vegan package. Multiple pairwise PERMANOVA in *adonis2* was also used to analyze the spatial variation in the root AMF community of each host species (tree and herb). The average relative abundance of each OTU at each microsite was calculated (*n* = 5). Then, the average value of each OTU across the four microsites was calculated to represent the average relative abundance of OTUs at the study site (*n* = 4). A heatmap of the major OTUs (those with an average relative abundance > 1% at the study site) was visualized to summarize the bipartite AMF networks of *Cr. japonica* trees and *Ch. serratus* herbs sets, with a special focus on the OTUs that were simultaneously dominant (those with a relative abundance > 10%) in their roots at the same microsite. We also analyzed the relationships between the relative abundance of OTUs and the physicochemical environment using redundancy analysis (RDA) with PERMANOVA.

## Results

### Soil physicochemical properties

All soil physicochemical properties, except EC, differed significantly among the microsites (Tables 1 and S1). pH was significantly different between MS1 and the other microsites. Total C and N were not significantly different between microsites located on the same topographic profile, that is, MSs 1 and 2 vs. MSs 4 and 5; however, the difference was significant compared with microsites located on the opposite topographic profile.

### Sequencing summary

We obtained 1,150,017 amplicon sequences which clustered into 10,513 OTUs at 97% sequence similarity with more than 8,955 reads per sample (maximum of 47,150) after trimming, pair joining, and quality filtering. After the removal of chimeric OTUs, non-Glomeromycotina OTUs, and rare OTUs, 984,182 sequences remained, which were clustered into 683 OTUs, with the minimum and maximum numbers of filtered sequences per sample being 7,625 and 24,604, respectively.

### Composition and spatial variation patterns of AMF communities in the roots of *Cr. japonica* and *Ch. serratus*

We recovered 670 and 679 AMF OTUs from the roots of *Cr. japonica* and *Ch. serratus*, respectively (Online Resource 3). We detected 17 major OTUs (average relative abundance > 1% at the study site), belonging to *Acaulospora* (1 OTU), *Dominikia* (2), *Glomus* (8), *Microkamienskia* (2), *Rhizophagus* (2), *Septoglomus* (1), and *Sclerocystis* (1), in the roots of both host plant species (Table 2, Online Resource 4). The microsite but not host plant species significantly affected OTU richness and Shannon index (Tables 3 and S2). The average OTU richness ranged from 246 to 301 and 201 to 303 in *Ch. serratus* herbs and *Cr. japonica* trees, respectively. The average OTU richness significantly varied between microsites in *Cr. japonica* trees (ANOVA: *F* = 7.92, *p* < 0.002) but not *Cr. serratus* herbs (ANOVA: *F* = 2.72, *p* = 0.119). However, the Shannon index did not significantly vary between microsites in neither host plant species but the magnitude of variation was higher in the tree (ANOVA: *F* = 2.86, *p* = 0.07) than the herb (ANOVA: *F* = 0.99, *p* = 0.42).

**Table 2:** Average relative abundance of major arbuscular mycorrhizal fungi (AMF) in the roots of *Cryptomeria japonica* and *Chloranthus serratus* at different microsite (MS) of the study forest

Accession of the AMF OTUs	Genus	*Cryptomeria japonica*				*Chloranthus serratus*				Site average
		MS1	MS2	MS4	MS5	MS1	MS2	MS4	MS5	
OR740684*	*Dominikia*	0.301	0.269	0.137	0.278	0.262	0.067	0.292	0.250	0.232
OR740685*	*Microkamienskia*	0.050	0.221	0.027	0.042	0.147	0.137	0.033	0.057	0.089
OR740686*	*Rhizophagus*	0.054	0.033	0.048	0.088	0.085	0.032	0.182	0.038	0.070
OR740688*	*Glomus*	0.025	0.052	0.033	0.054	0.042	0.030	0.092	0.142	0.059
OR740687	*Dominikia*	0.074	0.015	0.037	0.063	0.089	0.011	0.074	0.077	0.055
OR740690*	*Glomus*	0.019	0.007	0.234	0.040	0.004	0.011	0.004	0.016	0.042
OR740689*	*Septoglomus*	0.023	0.022	0.013	0.041	0.011	0.164	0.013	0.019	0.038
OR740691*	*Glomus*	0.100	0.009	0.058	0.065	0.009	0.011	0.014	0.026	0.036
OR740694*	*Glomus*	0.001	0.010	0.137	0.003	0.008	0.000	0.009	0.012	0.023
OR740692*	*Glomus*	0.009	0.021	0.010	0.005	0.002	0.108	0.002	0.012	0.021
OR740695	*Rhizophagus*	0.042	0.011	0.034	0.040	0.003	0.006	0.015	0.008	0.020
OR740693	*Glomus*	0.008	0.018	0.010	0.004	0.001	0.094	0.002	0.011	0.019
OR740696	*Acaulospora*	0.007	0.057	0.004	0.007	0.005	0.049	0.004	0.006	0.017
OR740697	*Glomus*	0.016	0.005	0.018	0.021	0.014	0.006	0.023	0.033	0.017
OR740699	*Sclerocystis*	0.010	0.068	0.002	0.004	0.007	0.002	0.005	0.005	0.013
OR740698	*Microkamienskia*	0.010	0.004	0.005	0.014	0.031	0.004	0.017	0.010	0.012
OR740700	*Glomus*	0.015	0.003	0.002	0.006	0.025	0.003	0.006	0.024	0.010

OTUs, operational taxonomic units. We defined major OTUs as those with at least 1% of the average relative abundance at the study site. The the average relative abundance of each OTU in the roots of five trees at each microsite was calculated, then averaged across the four microsites to represent the average relative abundance of the OTUs at the study site. Asterisks following the accessions refer to the dominant OTUs (average relative abundance in roots at the site or microsite > 10%). The table is sorted by the column "Site average"

**Table 3:** Alpha diversity of arbuscular mycorrhizal fungi communities in roots of nearby *Cryptomeria japonica* trees and *Chloranthus serratus* herbs

Microsite (MS)	Pairs of *Cr. japonica* and *Ch. serratus*	Number of OTUs		Shannon index	
		*Cr. japonica*	*Ch. serratus*	*Cr. japonica*	*Ch. serratus*
MS1	P01–P05	303 ± 20	301 ± 18	2.89 ± 0.16	2.8 ± 0.26
MS2	P08–P12	201 ± 36	246 ± 27	2.38 ± 0.2	2.74 ± 0.24
MS4	P22–P26	223 ± 28	260 ± 50	2.49 ± 0.52	2.66 ± 0.28
MS5	P15–P19	245 ± 38	271 ± 17	2.85 ± 0.17	2.96 ± 0.22

Values are presented as average ± SD (*n* = 5). Microsite and host plant species but not their interaction affected the number of OTUs. Only microsite had a significant effect on the Shannon index (Table S2)

**Fig. 1:**
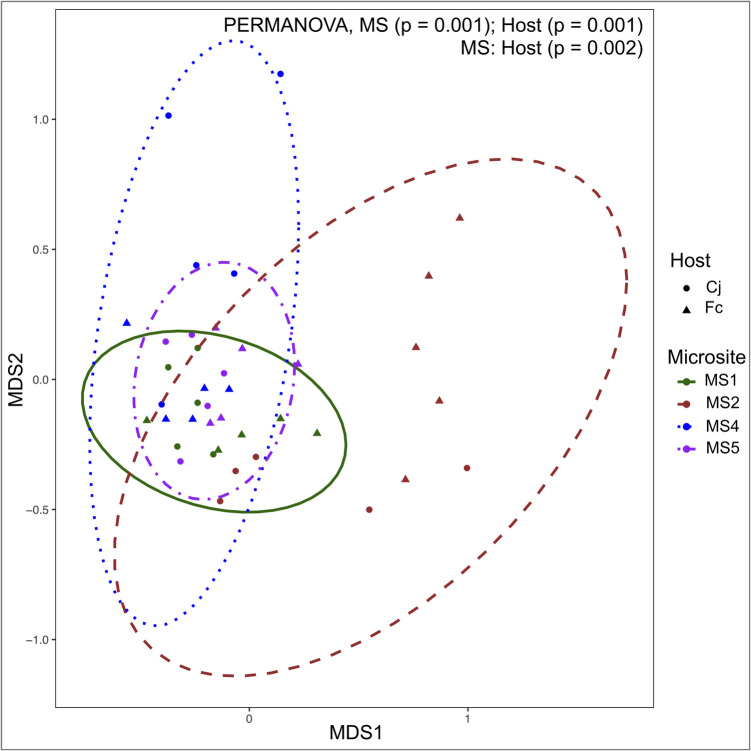
Non-metric multidimensional scaling of the arbuscular mycorrhizal fungi communities in roots of nearby *Cryptomeria japonica* (Cj) trees and *Chloranthus serratus* (Cs) herbs. Stress = 0.18

Microsite (*F* = 4.06, *p* < 0.001), host plant species (*F* = 3.19, *p* < 0.001), and their interaction (*F* = 2.26, *p* = 0.001) significantly explained the observed dispersion of samples (spatial variation in each host plant species and variation between host plant species) based on the AMF community composition (Fig. 1, Tables S3, and S4). The AMF communities in *Cr. japonica* and *Ch. serratus* were significantly different at MS2 (ANOSIM, *R* = 0.52, *p* = 0.02) and MS4 (*R* = 0.40, *p* = 0.01), but not at MS1 (*R* = 0.28, *p* = 0.05) and MS5 (*R* = 0.09, *p* = 0.27) (Fig. 1).

**Fig. 2:**
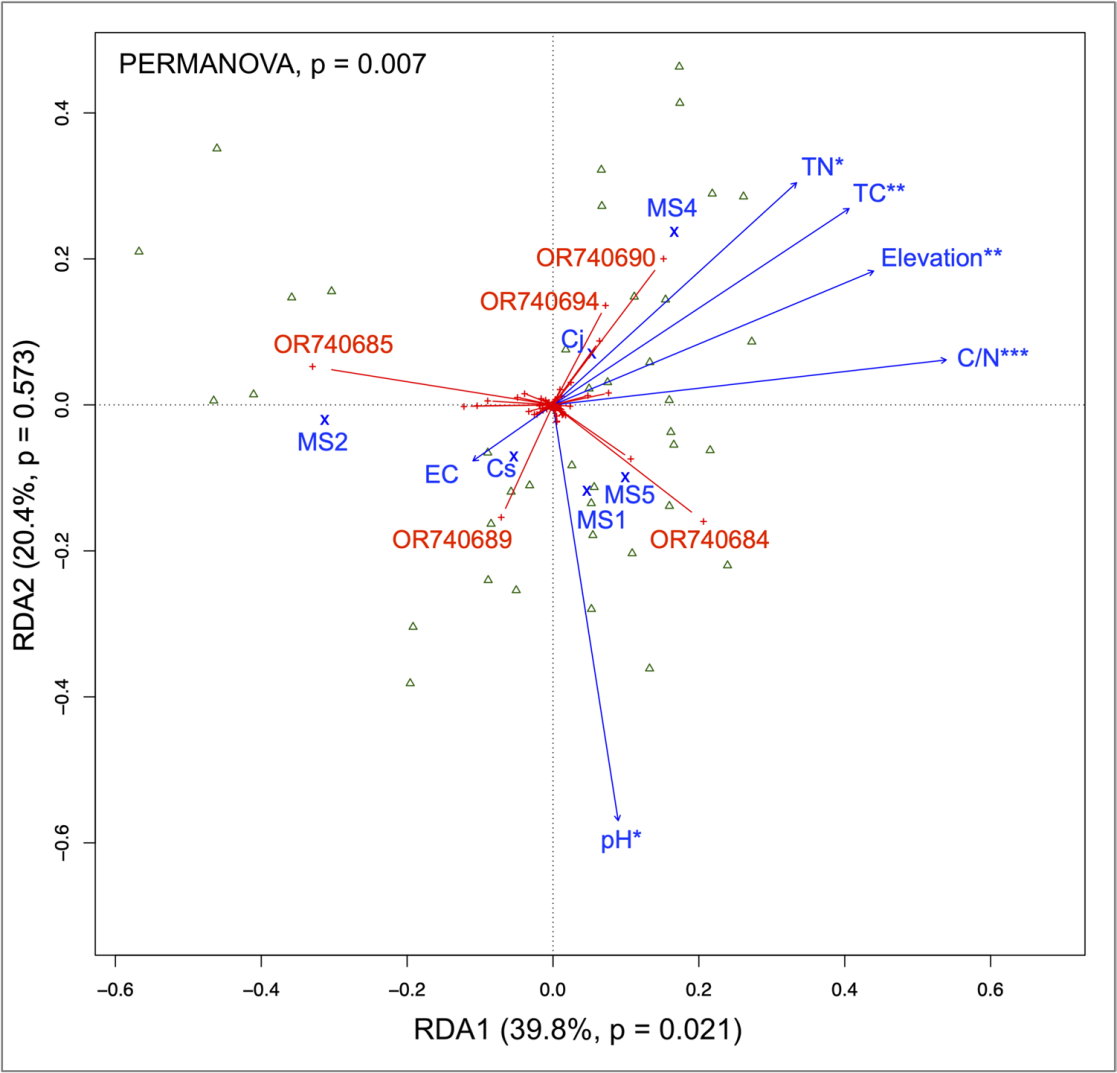
Triplots of redundancy analysis (RDA) of the arbuscular mycorrhizal fungi (AMF) communities in roots of nearby *Cryptomeria japonica* (Cj) trees and *Chloranthus serratus* (Cs) herbs, visualized on scaling 2. Asterisks following the names of vectors indicate a significant correlation with the AMF community composition (***, *p* < 0.001; **, *p* < 0.01; *, *p* < 0.05). OR###### are the accessions of the five operational taxonomic units most influenced by soil physicochemical properties, explaining the variation observed in the AMF community; MS, microsite; TC, total C; TN, total N; C/N, carbon to nitrogen ratio; EC, electroconductivity

RDA indicated significant linear relationships between AMF in the community and the environmental variables (Permutational test on RDA: *F* = 1.56, *p* = 0.01). The first axis was significant (RDA1: *F* = 6.20, *p* = 0.02), explaining 39.8% of the total variance across the microsites (goodness of fit, *p* < 0.001). The second axis (RDA2: *F* = 3.17, *p* = 0.57), which represented variations over the host species, was not significant (goodness of fit, *p* = 0.17, Fig. 2). On the first axis, C/N (*p* = 0.001, *R*^*2*^ = 0.34), elevation (*p* < 0.005, *R*^*2*^ = 0.26), total C (*p* < 0.005, *R*^*2*^ = 0.26), pH (*p* = 0.012, *R*^*2*^ = 0.21), and total N (*p* = 0.01, *R*^*2*^ = 0.21) but not EC (*p* = 0.68, *R*^*2*^ = 0.02) were detected to ordinate the AMF OTUs. The linear relationships were the strongest for 35 AMF OTUs (*r*^*2*^ > 50%, *p* = 0.01; Table S5). Among them, OR740684 (*Dominikia*, significantly associated with MSs 1 and 5), OR740685 (*Microkamienskia*, MS2), OR740689 (*Septoglomus*, MS2), OR740690 (*Glomus*, MS4), and OR740694 (*Glomus*, MS4) were the five OTUs most affected by soil physicochemical properties (Fig. 2, Tables 2 and S5). The two most abundant AMF in *Cr. japonica* and *Ch. serratus* were negatively correlated, showing different relationships with soil pH (Fig. 2).

**Fig. 3:**
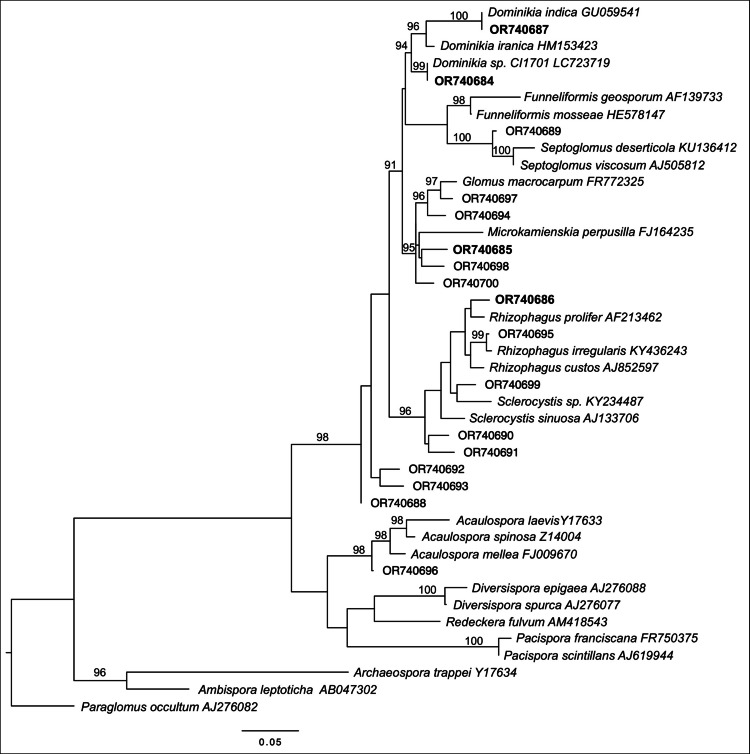
Phylogenetic tree of the major operational taxonomic units (OTUs) of arbuscular mycorrhizal fungi (AMF) in the roots of nearby *Cryptomeria japonica* and *Chloranthus serratus*. Major OTUs are defined as those with at least 1% of average relative abundance at the study site. A maximum likelihood tree was constructed using the representative sequences of the major OTUs (17 nucleotide sequences) and 25 reference nucleotide sequences downloaded from NCBI GenBank and Maarj*AM* databases. Aligned sequences had ~ 550 bp of the small subunit ribosomal DNA between the primer pairs NS31 and AM1 and covered 560 sites. The best model and parameters were selected with an automatic model finder in IQ-TREE 2. Ultrafast bootstrap (UFBoot) over 1000 randomizations were performed and UFboot ≥ 90% are shown at the nodes. Accessions of the major OTUs and scientific names of reference sequences followed by their accessions were used for labeling. The simultaneously dominant OTUs in nearby *Cr. japonica* and *Ch. serratus* are in bold

Regardless of the similarity or dissimilarity in AMF communities between *Cr. japonica* and *Ch. serratus* at different microsites, four dominant AMF OTUs (OR740684, *Dominikia*; OR740685, *Microkamienskia*; OR740686, *Rhizophagus*; and OR740687, *Dominikia*) were abundant (relative abundance > 10%) in 17 paired roots of *Cr. japonica* and *Ch. serratus* (Table 4, Fig. 3, Online Resource 4).

**Table 4:** Simultaneously dominant arbuscular mycorrhizal fungi in *Cryptomeria japonica* tree–*Chloranthus serratus* herb pairs

Tree–Herb pairs	OR740684		OR740685		OR740686		OR740687	
	*Dominikia*		*Microkamienskia*		*Rhizophagus*		*Dominikia*	
MS1P01	0.39	0.34						
MS1P02	0.20	0.29			0.22	0.36		
MS1P03	0.20	0.37						
MS1P04	0.30	0.12					0.11	0.20
MS1P05	0.41	0.19						
MS2P09			0.27	0.10				
MS2P10			0.13	0.18				
MS2P11	0.34	0.10						
MS2P12			0.57	0.33				
MS4P22								
MS4P23	0.16	0.26						
MS4P24	0.13	0.16						
MS4P26	0.30	0.35						
MS5P15	0.27	0.35					0.11	0.12
MS5P16	0.42	0.17						
MS5P17	0.20	0.13						
MS5P18	0.16	0.42						
MS5P19	0.33	0.18						

The table shows the relative abundance of the dominant operational taxonomic units (OTUs with at least 10% relative abundance) in the roots of *Cr. japonica* (left) and *Ch. serratus* (right). MSXPY refers to the Y pair of *Cr. japonica* and *Ch. serratus* root samples from microsite X. Empty cells refer to values < 10% (not a dominant OTU in the corresponding *Cr. japonica* and *Ch. serratus* pair). The full occurrence patterns of all major OTUs for all 20 pairs of samples are available in Online Resource 4

## Discussion

Mycorrhizal associations in Chloranthaceae plant species, such as *Ch. serratus*, have been poorly investigated, and information on their associated AMF is lacking (Wang and Qiu 2006). We determined the composition of the AMF communities in the roots of *Cr. japonica* trees and nearby *Ch. serratus* herbs. Our first record of mycorrhizal associations revealed an average of 210–310 AMF OTUs in the roots of *Ch. serratus*, and the dominant AMF belonging to *Dominikia* (OR740684 and OR740687), *Microkamienskia* (OR740685), and *Rhizophagus* (OR740686) simultaneously and abundantly colonize the roots of nearby *Cr. japonica*. We also found significant spatiality in the similarity of AMF communities between the roots of *Cr. japonica* and the roots of *Ch. serratus*, as explained by soil physicochemical properties. Also, the alpha diversity of root AMF communities in *Cr. japonica* trees was spatially and significantly varied while that in *Ch. serratu*s herbs was not. The investigated trees and herbs were growing adjacent to each other, but we observed different spatial variation patterns in their root colonizing AMF communities. The findings suggest that different mechanisms drive the response of root-colonizing AMF communities in trees and neighboring herbs to changes in soil physicochemical conditions. The observed within-site spatial variation in the AMF communities associated with *Cr. japonica* trees and *Ch. serratus* herbs were explained by the strong and significant influences of elevation, pH, total C, and N, but not EC, especially on five AMF belonging to *Dominikia* (OR740684), *Microkamienskia* (OR740685), *Septoglomus* (OR740689), and *Glomus* (OR740690 and OR740694). The AMF communities recovered from nearby *Cr. japonica* trees and *Ch. serratus* herbs varied spatially, explained by the distribution of their dominant AMF OTUs across microsites. This finding suggests that soil properties can filter AMF communities, which may, in turn, be reflected in the communities in neighboring *Cr. japonica* and *Ch. serratus*.

The co-occurring trees and herbs investigated in the current study differed in their root AMF community composition at two microsites but did not at two others, showing strong association with soil physicochemical properties. However, the two host plant species associated with approximately the same number of AMF species regardless of the microsite. A previous study on AMF communities in two co-occurring plants, an annual and a perennial herb, also reported different root colonizing AMF communities but a higher AMF richness in the annual plant species than in the perennial plant species (Torrecillas et al. 2012). Trees of *Cr. japonica* and *Chamaecyparis obtusa* (both belonging to Cupressaceae) in proximity have been reported to have significantly similar root colonizing AMF communities although those in their surrounding soils were significantly dissimilar (Djotan et al. 2023). Therefore, the investigation of the similarity between the root colonizing AMF communities of the three combinations of plant co-occurrence in forests (tree—tree, tree—herb, and herb—herb) is now telling us that many factors including host phylogeny, soil physicochemical properties, and the co-occurring life forms could predict the similarity between the root colonizing AMF communities of neighboring plants in forests. On the other hand, the detection of different numbers of OTUs among different life forms of host plants may be linked to differential sampling efforts of the root systems. Herbaceous plants can be easily excavated to study AMF in the roots, whereas only a small fraction of tree plants’ root systems can be collected, with perennial herbs in the between. Thus, the proportion of the investigated roots to the total root system should be considered when comparing the AMF communities detected in the root systems of host plants of different life forms.

The four simultaneously abundant AMF in the roots of *Cr. japonica* trees and *Ch. serratus* belonged to the genera *Dominikia*, *Microkamienskia*, and *Rhizophagus*. These results are consistent with previous findings by Djotan et al. (2023, 2024a, b) regarding the taxonomic affiliation of the dominant AMF OTUs in the roots of *Cr. japonica* in its artificial forests. OR740684 (*Dominikia*) was the dominant AMF in the roots of *Cr. japonica* and *Ch. serratus* pairs at all microsites. The same AMF was detected in the roots of an initial mycoheterotrophic plant, *Gentiana zollingeri* (thus involved in mycoheterotrophy), and described as one of the cheating-susceptible AMF (Kusakabe and Yamato 2023). It has been suggested that partially mycoheterotrophic plants rely on neighboring autotrophic plants for nutrient acquisition via transfer with the mycelia of connecting mycorrhizal fungi (Cameron et al. 2009; Yamato et al. 2023; Sakae et al. 2024). The trees and herbs investigated here were both autotrophic and four AMF OTUs including OR740684 (*Dominikia*) were simultaneously abundant in nearby individuals. We found that its nucleotide sequence was identical to that of an AMF isolate, likely to be *Dominikia aurea* CI1701 registered under the accession number LC723719 (Kusakabe and Yamato 2023). This AMF corresponds to VTX00166, which has a global distribution in various ecosystems and has also been detected in the roots of mycoheterotrophic plants belonging to many families (e.g., Suetsugu et al. 2022; Kusakabe and Yamato 2023). To the best of our knowledge, our study is the first to report that an identical OTU of AMF is dominant and shared in the roots of neighboring autotrophic plants, i.e., *Cr. japonica* trees and *Ch. serratus* herbs. This simultaneously dominant AMF detected in the roots of the nearby *Cr. japonica* trees and *Ch. serratu*s herbs could be involved in nutrient transfer between the two host plant species and other mycoheterotrophic plants. The three other abundant AMF OTUs have not been reported to be involved in mycoheterotrophy. The OTU OR740687 (*Dominikia* sp.), which belongs to VTX00222, was identical to *Dominikia indica* (synonym *Glomus indicum*, GU059541; Błaszkowski et al. 2010). Although the OTU OR740685 and OTU OR740686 did not match any virtual taxa, they were nearly related to *Microkamienskia perpusilla* (FJ164235; Błaszkowski et al. 2009; Corazon-Guivin et al. 2019) and *Rhizophagus prolifer* (AF213462; Declerck et al. 2000; Schüßler and Walker 2010), respectively in the phylogeny.

In the current study, the variation in pH, total C, and total N among the microsites was unrelated to their positions on the topographic profile (slope top, middle, and downslope). However, the microsites on the same topographic profile had similar total C and N but differed significantly from those on the opposite profile. These observed variation patterns, which were similar to our findings in a previous study at the same microsites (Djotan et al. 2024b), may indicate that spatial variations in soil physicochemical properties in planted forests of *Cr. japonica* were related to the microbial community and factors such as bedrock, slope, and vegetation cover rather than to their position on the topographic profiles.

In conclusion, we partially validated the hypothesis that *Cr. japonica* and *Ch. serratus* associate with similar AMF communities when growing adjacent to each other. The similarity of their associated AMF communities varied spatially, explained by the microsite-specific distribution of the dominant AMF in their roots. The dominant *Dominikia*, *Microkamienskia*, and *Rhizophagus* abundantly colonized the roots of neighboring *Cr. japonica* trees and *Ch. serratus* herbs. Four AMF OTUs were simultaneously dominant in pairs of host plant species, suggesting the sharing of the same AMF species, opening the doors to nutrient flow studies.

## Supplementary Information

Below is the link to the electronic supplementary material.

Supplementary file1 (DOCX 6663 KB)

## Data Availability

We deposited the sequence read archives at the NCBI (PRJNA898865). The representative nucleotide sequences of the AMF OTUs generated (OR740684–OR741366, SUB13934051) and a representative partial nucleotide sequence of *rbc*L for *Cr. japonica* (OP832014, BankIt 2642437) and *Ch. serratus* (OP832018, BankIt 2642437) were submitted to GenBank.
