# Supplementary material for: The similarity between arbuscular mycorrhizal fungi communities of trees and nearby herbs in a planted forest exhibited within-site spatial variation patterns explained by local soil conditions
Source: Mycorrhiza. 2025 Mar 14;35(2):21. doi: 10.1007/s00572-025-01197-5 (PMC11909092; doi:10.1007/s00572-025-01197-5)
Supplement: Supplementary file 1 — Supplementary file1 (DOCX 6663 KB) [file 572_2025_1197_MOESM1_ESM.docx]

**The similarity between arbuscular mycorrhizal fungi communities of trees and nearby herbs in a planted forest exhibited within-site spatial variation patterns explained by local soil conditions**

Akotchiffor Kevin Geoffroy Djotan ^*1,3^, Norihisa Matsushita ^*2,4^, Yosuke Matsuda ^1,5^, Kenji Fukuda ^2,6^

^1^University of Tokyo, Graduate School of Agricultural and Life Sciences, ^2^Mie University, Graduate School of Bioresources

ORCID: ^3^https://orcid.org/0000-0002-3726-9826; ^4^https://orcid.org/0000-0003-3281-8846, ^5^https://orcid.org/0000-0002-7001-3101, ^6^https://orcid.org/0000-0002-9980-3107

*^*^Corresponding author, E-mail: geoffroydjotan@yahoo.fr*, *nmatsushita@g.ecc.u-tokyo.ac.jp*

**Supplementary materials**


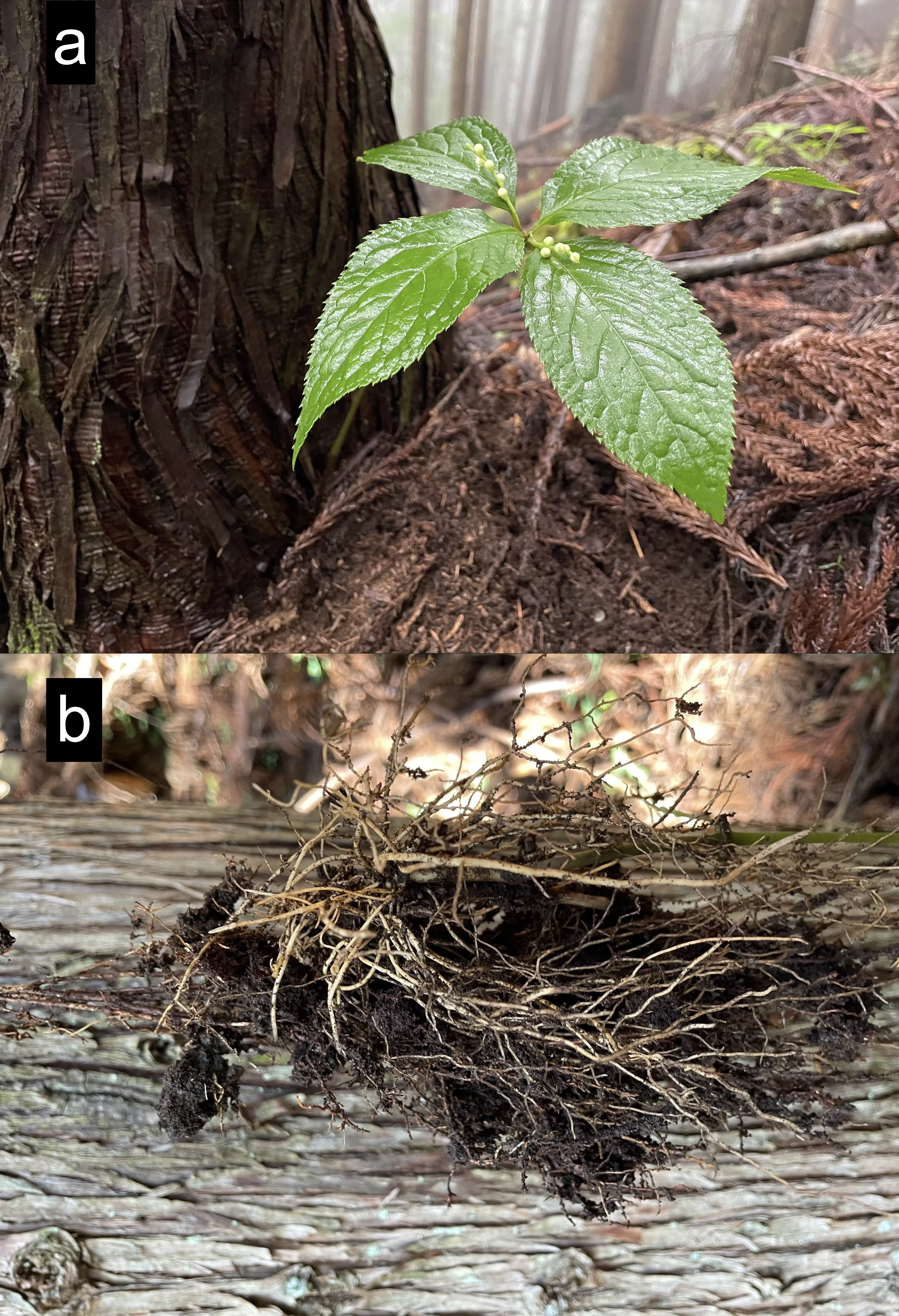


Online Resource 1 A plant of *Chloranthus serratus* growing near a tree of *Cryptomeria japonica* (a) and extremely interwoven roots of both species (b)


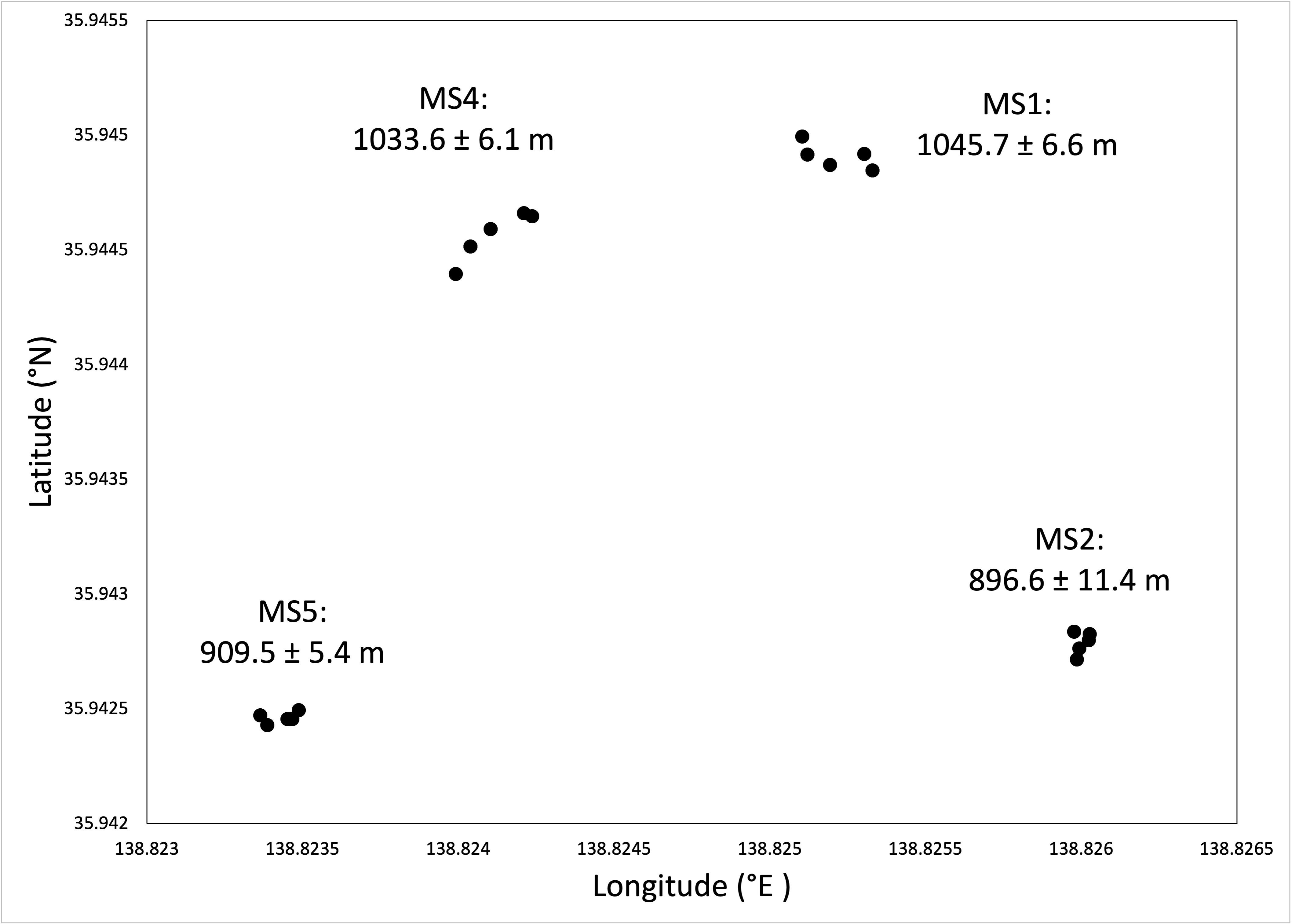


Online Resource 2 Distribution of the sampled pairs of nearby Cryptomeria japonica and Chloranthus serratus. Each point represents a pair of the host at the corresponding microsite. Values indicate the elevation (average ± SD) at the microsite (MS)


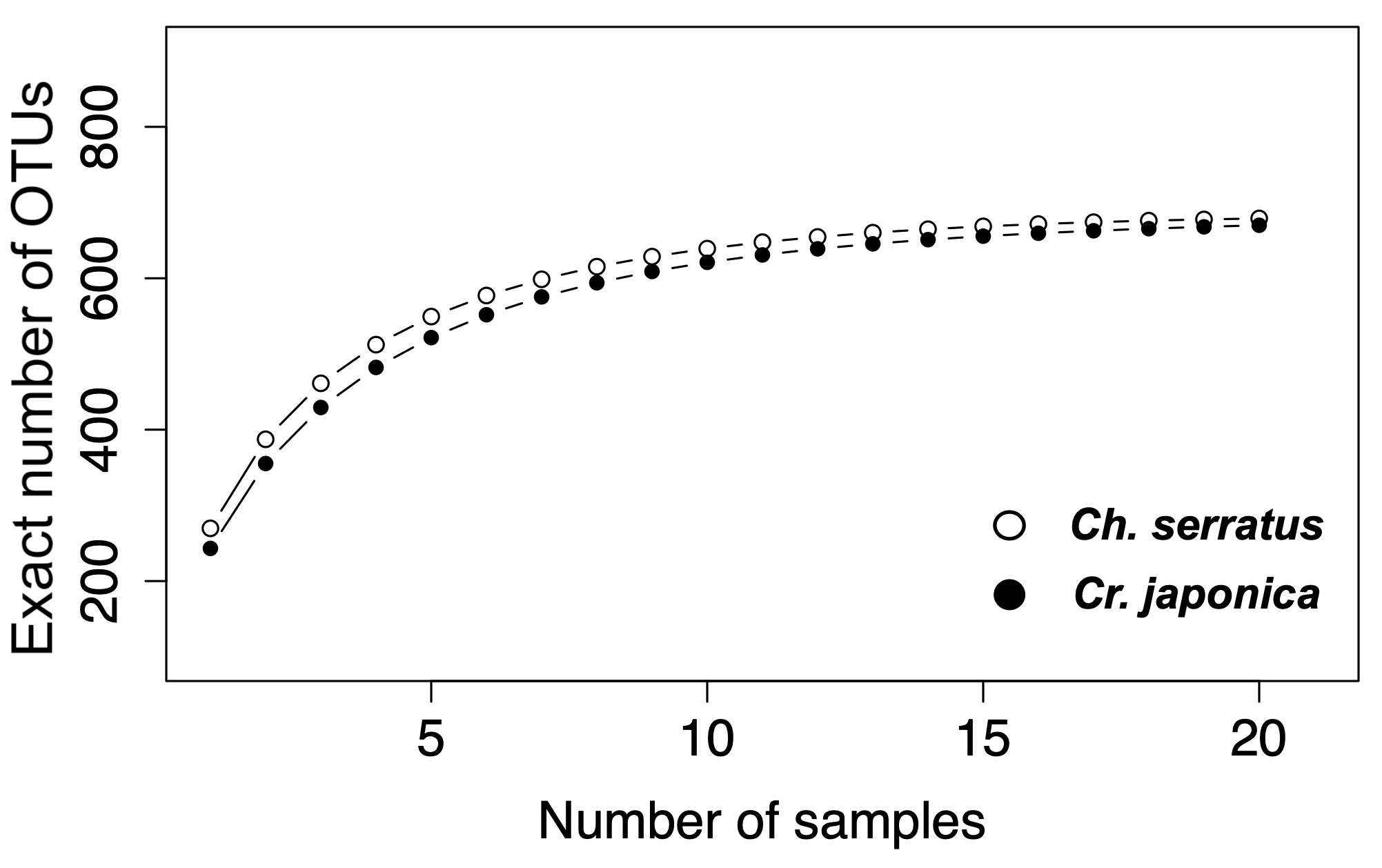


Online Resource 3 Accumulation curves of the operational taxonomic units of arbuscular mycorrhizal fungi (AMF) in the roots of nearby Cryptomeria japonica and Chloranthus serratus


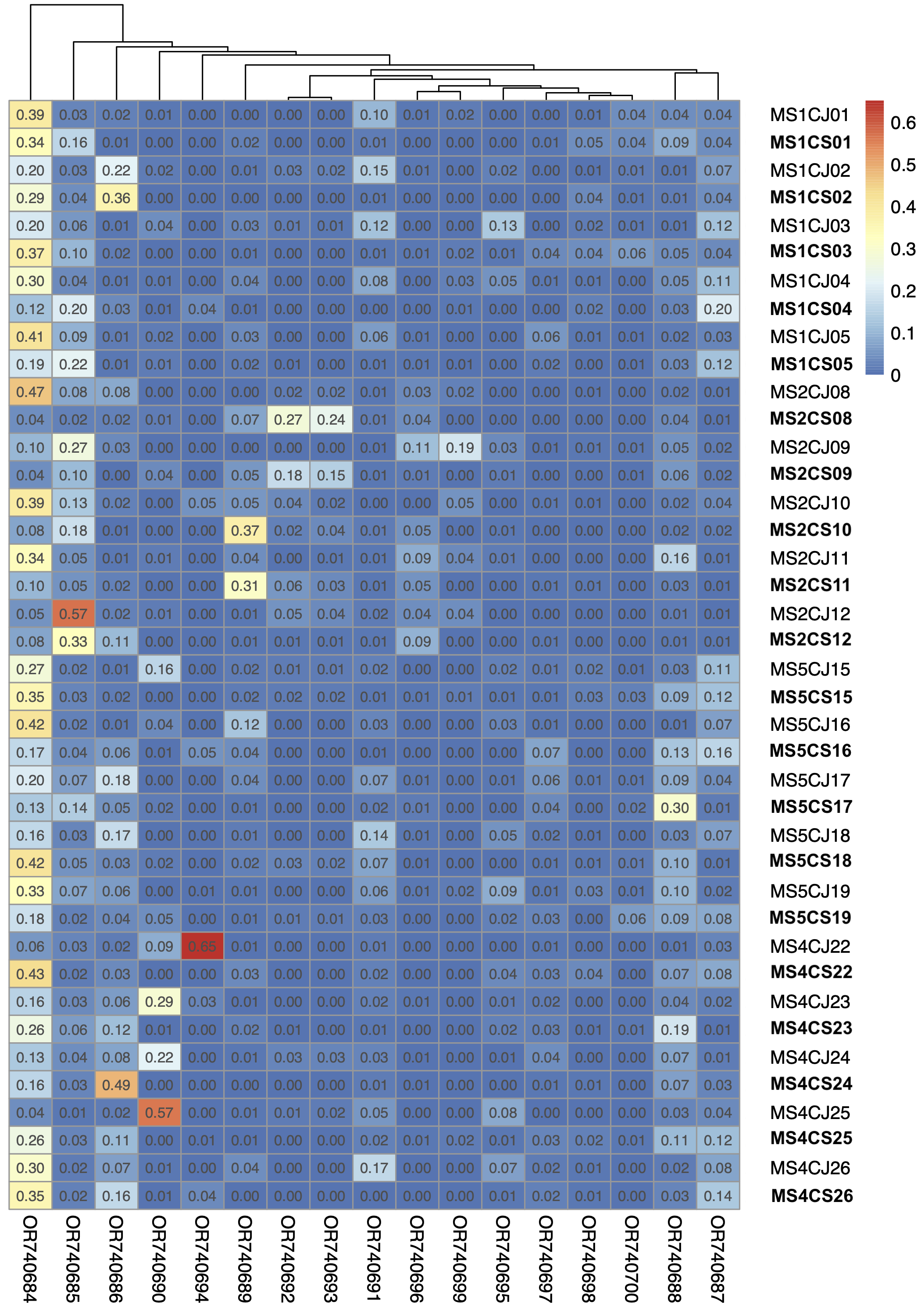


Online Resource 4 Distribution of the major operational taxonomic units of arbuscular mycorrhizal fungi in the roots of nearby Cryptomeria japonica (CJ) and Chloranthus serratus (CS). The first three characters in the sample labels indicate the microsite (MS#), and the following two indicate the host species (CJ and CS). The last two indicate the positions at the microsite. Labels are bold for CS’s samples

Table S1 Test of spatial variations in the soil's physicochemical properties and elevation

| Soil's physicochemical properties | Test | *p* value ^a)^ |
| --- | --- | --- |
| Electroconductivity (EC) | Analysis of variance (ANOVA) | 0.49 |
| Elevation | ANOVA | < 0.001 |
| pH | ANOVA | < 0.001 |
| Total C (TC) | ANOVA | < 0.001 |
| Total N (TN) | ANOVA | < 0.001 |
| Total C/N | Kruskal-Wallis rank sum | < 0.001 |

^a)^ Significant variations reflected by p-value < 0.05

Table S2 Analysis of variance on alpha diversity indices of arbuscular mycorrhizal fungi community in roots of nearby Cryptomeria japonica and Chloranthus serratus

| Variable & Factor ^a)^ | Df | Sum Sq | Mean Sq | F value | Pr(>F) |
| --- | --- | --- | --- | --- | --- |
| Number of OTUs |  |  |  |  |  |
| Microsite | 3 | 33998 | 11333 | 9.36 | < 0.001 |
| Microsite:Host | 4 | 10261 | 2565 | 2.12 | 0.10 |
| Residuals | 32 | 38727 | 1210 |  |  |
| Shannon index |  |  |  |  |  |
| Microsite | 3 | 0.9509 | 0.317 | 3.30 | 0.03 |
| Microsite:Host | 4 | 0.4422 | 0.1105 | 1.15 | 0.35 |
| Residuals | 32 | 3.0721 | 0.096 |  |  |

^a)^ Variables are the number of operational taxonomic units (OTU) and the Shannon index. Factors are Microsite and Host. Pr(>F) < 0.05 shows a significant effect of the corresponding factor on the variable

Table S3 Permutational analyses of variance on the arbuscular mycorrhizal fungi communities in roots of nearby Cryptomeria japonica and Chloranthus serratus

| A: All hosts | | | | | |
| --- | --- | --- | --- | --- | --- |
| Factor | Df | SumOfSqs | R2 | F | Pr(>F) |
| Microsite | 3 | 1.5476 | 0.22508 | 4.06 | < 0.001 |
| Microsite:Host | 4 | 1.2655 | 0.18406 | 2.49 | < 0.001 |
| Residual | 32 | 4.0625 | 0.59086 |  |  |
| Total | 39 | 6.8756 | 1 |  |  |
| B: *Cryptomeria japonica* only | | | | | |
| Factor | Df | SumOfSqs | R2 | F | Pr(>F) |
| Microsite | 3 | 1.0587 | 0.32842 | 2.6082 | < 0.001 |
| Residual | 16 | 2.1649 | 0.67158 |  |  |
| Total | 19 | 3.2237 | 1 |  |  |
| C: *Chloranthus serratus* only | | | | | |
| Factor | Df | SumOfSqs | R2 | F | Pr(>F) |
| Microsite | 3 | 0.6349 | 0.19695 | 1.308 | < 0.001 |
| Residual | 16 | 2.5888 | 0.80305 |  |  |
| Total | 19 | 3.2237 | 1 |  |  |

Table S4 Probabilities associated with multiple pairwise PERMANOVA of the root arbuscular mycorrhizal fungi communities of nearby Cryptomeria japonica (lower left) and Chloranthus serratus (upper right) between microsites

| Microsites | MS1 | MS2 | MS4 | MS5 |
| --- | --- | --- | --- | --- |
| MS1 |  | 0.01 | 0.10 | 0.10 |
| MS2 | 0.01 |  | 0.01 | < 0.001 |
| MS4 | 0.04 | 0.01 |  | 0.23 |
| MS5 | 0.55 | 0.01 | 0.10 |  |

p-value < 0.05 indicates significantly dissimilar AMF assemblages between the tree and the herb at the microsite

Table S5 Operational taxonomic units (OTUs) with significant contributions to the variation of the arbuscular mycorrhizal fungi communities in the roots of nearby Cryptomeria japonica and Chloranthus serratus

| OTUs | r^2^ | Pr(>r) |
| --- | --- | --- |
| OR740684 | 0.780 | 0.001 |
| OR740685 | 0.708 | 0.001 |
| OR740947 | 0.631 | 0.001 |
| OR740858 | 0.586 | 0.001 |
| OR740957 | 0.570 | 0.001 |
| OR740690 | 0.568 | 0.001 |
| OR740765 | 0.544 | 0.001 |
| OR740907 | 0.543 | 0.001 |
| OR740786 | 0.542 | 0.001 |
| OR741133 | 0.519 | 0.001 |
| OR740809 | 0.511 | 0.001 |
| OR740922 | 0.478 | 0.001 |
| OR740924 | 0.425 | 0.001 |
| OR741080 | 0.418 | 0.001 |
| OR740696 | 0.411 | 0.001 |
| OR741152 | 0.383 | 0.001 |
| OR741274 | 0.491 | 0.002 |
| OR741029 | 0.457 | 0.002 |
| OR741292 | 0.438 | 0.002 |
| OR740744 | 0.402 | 0.002 |
| OR741047 | 0.375 | 0.002 |
| OR740908 | 0.318 | 0.002 |
| OR740981 | 0.406 | 0.003 |
| OR740950 | 0.402 | 0.003 |
| OR740813 | 0.396 | 0.003 |
| OR740925 | 0.387 | 0.003 |
| OR740762 | 0.387 | 0.003 |
| OR740994 | 0.357 | 0.003 |
| OR741329 | 0.312 | 0.003 |
| OR740843 | 0.255 | 0.003 |
| OR741289 | 0.368 | 0.004 |
| OR740868 | 0.354 | 0.004 |
| OR741226 | 0.370 | 0.005 |
| OR740903 | 0.290 | 0.005 |
| OR740960 | 0.269 | 0.005 |
